# Comparative screening the life-time composition and crystallinity variation in gilthead seabream otoliths *Sparus aurata* from different marine environments

**DOI:** 10.1038/s41598-022-13667-3

**Published:** 2022-06-10

**Authors:** Geza Lazar, Fran Nekvapil, Sanja Matić-Skoko, Călin Firta, Dario Vrdoljak, Hana Uvanović, Lucian Barbu-Tudoran, Maria Suciu, Luka Glamuzina, Branko Glamuzina, Regina Mertz-Kraus, Simona Cinta Pinzaru

**Affiliations:** 1 Ioan Ursu Institute of Physics, Babes Bolyai University, Kogalniceanu 1, 400084 Cluj-Napoca, Romania; 2 Institute of Oceanography and Fisheries, Šetalište Ivana Meštrovića 63, 21000 Split, Croatia; 3 National Institute for Research and Development of Isotopic and Molecular Technologies, 67-103 Donat, 400293 Cluj-Napoca, Romania; 4 Department of Aquaculture, University of Dubrovnik, Ćira Carića 4, 20 000 Dubrovnik, Croatia; 5 Institute for Geosciences, Johannes Gutenberg University, Mainz, Germany

**Keywords:** Biochemistry, Ecology, Biogeochemistry, Environmental sciences

## Abstract

Differences in crystallinity, structure and composition variation along the growing direction in gilthead seabream, *Sparus aurata* otoliths that inhabited different environments were determined to evaluate the correlation of spectroscopic and chemical data with the lifetime development and movement pattern. The Raman spectroscopy signal provided the characteristic bands whose Full Width at Half Maximum (FWHM) were used to track the signal variability. The FWHM showed an initial increase in the core area, followed by a decrease depicting two minima coinciding growth rings. The crystal discontinuity linked to annual rings was confirmed. The FWHM pattern followed cycle in the individual’s activity. However, no significant correlation with FWHM and environmental factors although the slope of the FWHM variation distinguished aquaculture and costal groups from open sea and transitional, estuarine waters. Raman data were further correlated with morphological and elemental composition obtained via SEM–EDX and by LA-ICP-MS. SEM clearly confirmed CRM findings. Finally, multiparameter analysis of Ba/Ca concentrations obtained by LA-ICP-MS indicated the separation of groups associated with aquaculture and transitional waters due lowest variability in the elemental composition. Other groups are more variable possibly due to the water oligotrophic character and greater variability in prey availability in each environment. Results of the present study showed the additional potential of Raman spectroscopy as a complementary tool for inference of migration or origin of fish based on otolith composition and structure like other well-established technique.

## Introduction

Nowadays, otoliths becoming extremely valuable tools in revealing migration pathways and life-history traits of fishes. The term ‘migration’ is usually used for describing both small-scale spatial accidental changes and large-scale intentional movements from one region to another^1^. Irrespective of their spatial conditionality, it is still a very challenging to identify and describe migrations. Fish species can inhabit estuaries permanently, transitionally as anadromous and catadromous species or just occasionally like freshwater and marine stragglers or during certain life stages, as is the case of marine migrating juvenile fishes which use estuaries as essential nurseries^2^. Usually, such species have spatially segregated adults and juveniles where adults live in open marine environment and spawn offshore, larvae are transported towards the coast and post-larvae or early juveniles enter and settle in suitable nursery areas in shallow coastal zones. Otoliths grow continuously and record all changes in the environment that fish experience during their lifetime. Thus, the otolith core represents early life stages after hatching while the otolith edge corresponds to the state in the environment at the time of capture^1,3,4^. The potential cause-and-effect relationship between life spans and environmental changes has significantly increased interest in otolith biomineral research^1,2^. Otoliths are biogenic CaCO_3_ structures are isolated within a semi-permeable membrane and bathed in an endolymphatic fluid of the inner ear of the fish. They also have role in maintaining balance and for hearing^3,4^. The otolith is composed of a number of concentric rings with different radii creating a specific pattern in which the otolith nucleus is the first zone. Depending on the amount of organic material in each ring or zone, its appearance will vary from extremely opaque (dark) to completely hyaline (transparent). Generally, the opaque zones are formed during the period of greatest growth and the hyaline zones are laid down when growth is slowest. Previous work has demonstrated that otoliths of different fish species, can comprise two different CaCO_3_ crystalline structures, aragonite and vaterite^5–8^ which can differ dramatically in their trace elemental composition^9,10^. Tzeng et al.^11^ proved that failure in the identification of vaterite can lead to the misidentification of the environmental history of fish when elemental signatures are used as biological tracers. Micro-Raman spectrometry was used to investigate the formation of light and dark zones on otolith surface as well as the effects of staining and etching on the fractions of mineral and organic compounds found in the otolith^12^. Other techniques were used to determine the life history of the fish via otoliths such as laser ablation inductively coupled plasma mass spectroscopy^8,10,11,13,14^, laser ablation-multicollector inductively coupled mass spectroscopy^15^, wavelength-dispersive spectrometry^8^ and electron Microprobe^16^. In the marine environment, some elements like strontium and barium in various biological calcitic tissues have shown correlations with ocean water temperatures^17,18^. Exactly, strontium and barium are used successfully in reconstructing environmental and “costal-estuary” migration histories for individual fish^19^, as their concentrations reflects local availability in the seawater. Thus, it is well documented difference in the elemental ratios of otoliths with higher Sr found in marine and higher Ba found in freshwater^20^. A positive relationship between the Sr content of otoliths and ambient salinity has also been observed^21,22^.

Multiple recent studies have used micro-Raman spectroscopy to differentiate the calcium carbonate polymorphs^23–25^, however correlations between otolith growing feature, composition and morphology extracted from Raman spectroscopy information with those related to the environmental conditions along the fish lifetime are yet challenging. Most of the Raman studies of otoliths showed that characteristic polymorph of calcium carbonate biomatrix is aragonite with the typical Raman vibrational modes ν_1_ (1085 cm^−1^) and ν_4_ (701 cm^−1^ and 705 cm^−1^) as well as lattice modes (8 bands between 142 cm^−1^ and 282 cm^−1^)^26,27^. Some of the studies reported the coexistence of 3 polymorphs of calcium carbonate (calcite, aragonite, vaterite) in the growth rings of Antarctic bivalves having the same growing pattern as fish otoliths^16^, confirmed by Confocal Raman Microscopy (CRM). Concerning biological development, otolith growth is characterized by successive growing rings which are representing morphologically discontinuities of the crystal layers. Thus, Raman is usually used as validation method for annual growth ring formation^4^. Growing rings are only visible in transmission light microscopy, however previous studies showed that vaterite areas appear dark under reflected light after etching with EDTA (Ethylenediaminetetraacetic acid), thus it is an alternative method to differentiate aragonite from vaterite.

Our preliminary study on otolith microstructure using confocal Raman microscopy^27^ probed the potential of the technique to provide details related to the characteristic otolith growing pattern associated with the Raman signature, in terms of bands positions and width. We showed that a series of Raman spectra collected with a controlled lateral step of 50 μm from otolith core to margins, corresponding to the growing direction, invariably showed i) only aragonite signature; ii) slight variability in the relative intensity of the aragonite lattice modes; and iii) subtle changes in band positions and full width at half maximum (FWHM) on passing from otolith core to first ring and further to margins, indicating changes in crystallinity epitaxial growth. Moreover, spurious crystalline spots indicated random occurrence of trace of barium carbonate (witherite) or strontianite, as a result of Ba or Sr random intake along the fish lifetime, which clearly influenced the aragonite crystal growth. As such, the highly ordered crystalline polymorph was associated with the light rings (seen under optical microscopy) while the dark alternating rings were associated with less crystalline order suggesting non-uniform otolith crystal growth. These non-uniformities could be linked with the environmental factors, such as water chemistry, depth, temperature but also with physiological, intrinsic factors^13^ inherent to every individual and, therefore, require analyzes and comparisons on larger otolith groups.

The gilthead seabream, *Sparus aurata* is a commercially important fish species, both for the aquaculture and fisheries^28^. Recently, positive changes in species abundance and distribution were reported, implicating ocean warming and escape from aquaculture^29–31^ as potential factors behind increasing abundance^32–35^. Throughout the Mediterranean, it is known that *S. aurata* performs ontogenetic migrations related to spawning, settlement and recruitment between coastal lagoons and the sea^36–40^. Juveniles settle to the sheltered coastal areas in early winter and join adults in open sea the following autumn^41–43^. In the present study, differences in crystallinity variation along the growing direction in otolith cross section of gilthead seabream, *Sparus aurata* that inhabited open sea, coastal waters, estuary and aquaculture rearing cages, were extracted by the systematic investigation of the Raman spectra. The results we correlated with SEM–EDX data recorded from each otolith of the four groups and further with the LA-ICP-MS to evaluate lifetime development and to draw conclusions on any difference related to the local environment inhabited by each group. The main aim of this paper is to see whether the 3 complementary methods will offer specific signals in term of otolith composition, structure and crystallinity that will indicate different habitat origins.

## Results

**Figure 1:**
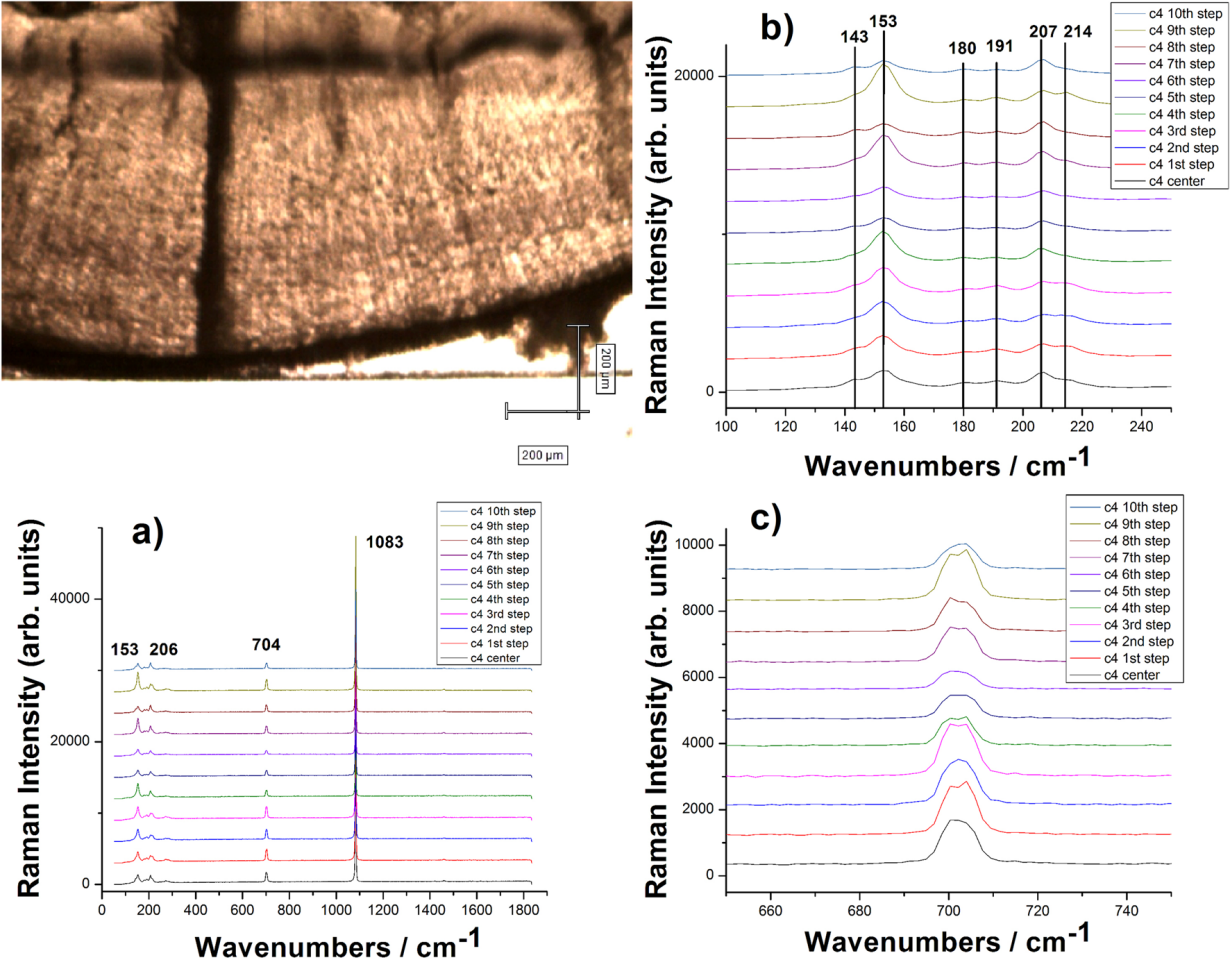
(**a**) Typical Raman spectra collected from one otolith (C4) core to margin using constant lateral step controlled from Raman instrument stage and a 20 X objective; (**b**, **c**) details of the spectral ranges containing the lattice modes (100–250 cm^−1^) and the bending mode around 700 cm^−1^ of aragonite respectively showing slight changes from one step to another. Color code for each consecutive step is indicated. Micrograph shows a fragment of the respective otolith where the second ring is clearly visible.

From a larger set of otoliths which we comprehensively investigated, we randomly selected one from the open sea for higher spatial investigation via CRM. Details of the otolith morphology and cross section along with indicated direction of analysis from the core towards the edge are presented in the Fig. 1. The bands observed in the Raman spectra indicate that the crystal structure is composed of aragonite^27^. We used the main Raman stretching mode of carbonate at 1083 cm^−1^ (ν_1_ CO_3_^2−^) as well as the bands from the 100–300 cm^−1^ region (lattice modes) characteristic for each polymorph of calcium carbonate to undoubtedly identify the polymorph in each otolith. The band at 701 cm^−1^ corresponds to the aragonite’s ν_4_ vibration mode, but the peak at 705 cm^−1^ is not visible thus appearing as a single peak instead of the doublet from the literature. Along with aragonite we found traces of calcite indicated by the band at 716 cm^−1^ (Fig. 1c). The other characteristic bands of calcite are covered by the strong aragonitic signal however the peak at 716 cm^−1^ was enough to identify calcite polymorph co-existence. No characteristic bands of vaterite were noted along the analysis of this particular otolith.

**Figure 2:**
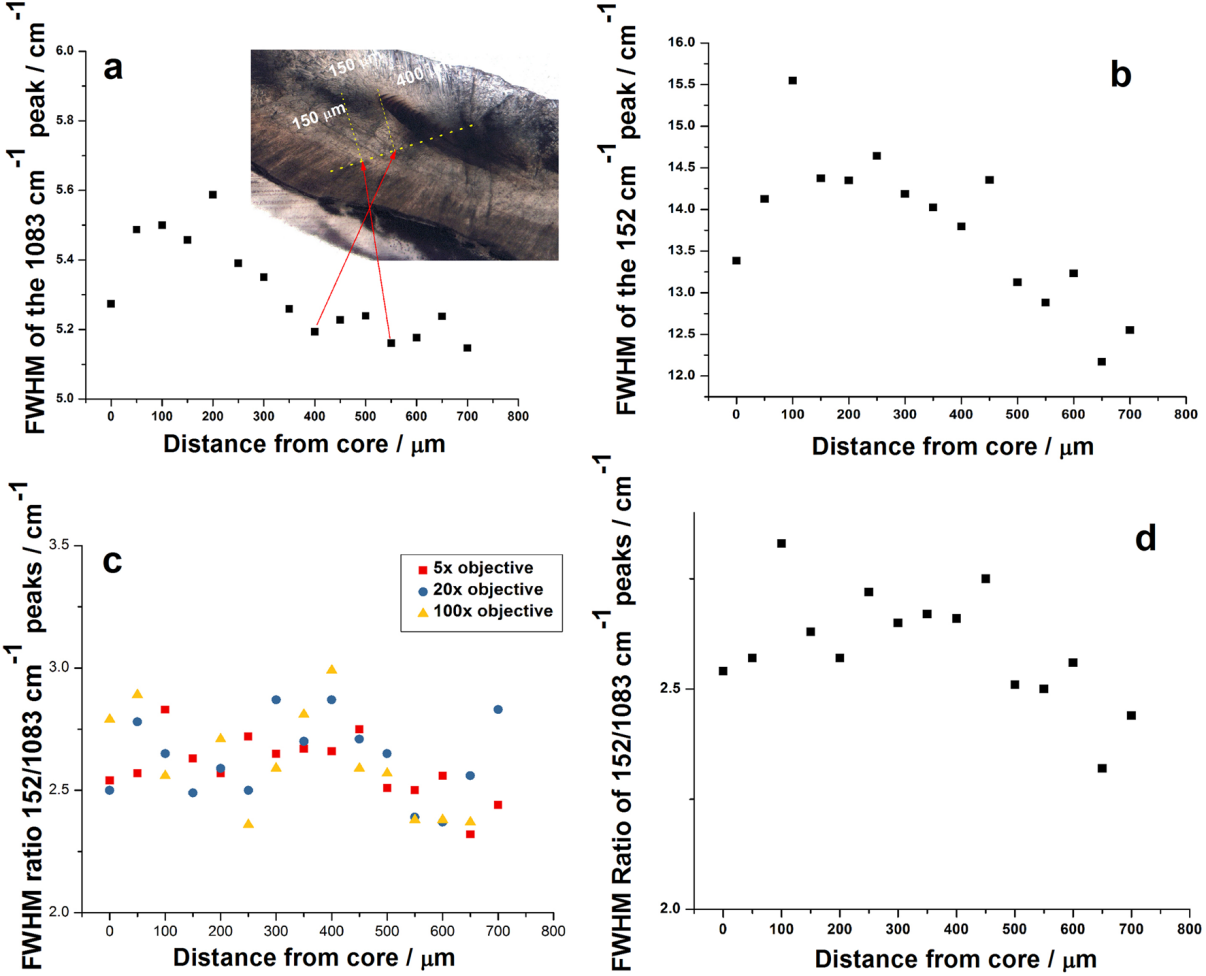
FWHM variation of the: (**a**) main carbonate Raman band (1083 cm^−1^), (**b**) lattice mode at 152 (cm^−1^), (**d**) the ratio of the 1083 and 152 cm^−1^ bands, along the growth line. (**c**) Comparison of the FWHM ratio (152/1083 cm^−1^), with different collecting objectives (5 ×, 20 × and 100 ×).

We calculated the FWHM for the main Raman bands from the series of spectra step scanned from the otolith from the core to margin. The higher the FWHM the lower the local crystallinity, revealed in the local crystal disorder. The evolution of these FWHM values extracted from the normalized and background subtracted spectra collected from the core to the edge with a 50 µm step were presented on Fig. 2. The display of the FWHM of the band at 1083 cm^−1^ along the growth line (Fig. 2a) is shown as well as the same variation for the FWHM of the lattice mode at 152 cm^−1^ (Fig. 2b). Considering that the collection optics influence the band broadening, we comparatively showed the FWHM values for three distinct collection objectives (Fig. 2c). The ratio between the FWHM values plotted in the Fig. 2a and b are presented in Fig. 2d. It is clearly noted that the crystallinity related to the sharpness of the Raman bands is variable along the crystal growth and the variability is not linear. The otolith margins tend to show the highest crystallinity.

An increase of the FWHM starting from core up to a 200 µm distance then a decrease along 550 µm and two minima (400 and 550 µm away from the core) are discernable at the distances in coincidence with the first and second ring respectively (Fig. 2a). This suggests that the crystalline structure is closest to ideal where discontinuities appear in growth. Also, the minimums correlated to winter season when slower growth is expected due to the lower sea temperature and shorter photoperiod. Similar trend has been observed in Fig. 2b and d with slightly larger differences. The 5 × objective was used due to its larger collecting spot and therefore better ability to eliminate errors and misidentifications. A comparative display of the evolution of the FWHM variation for the main stretching mode of aragonite (1083) using the 5 × 20 × and 100 × objectives was shown in Fig. 2c. The variability appeared consistent with lower values for the 100 × objective. Considering the experimental uncertainty as well as the broadening mechanism of Raman bands of crystals we note an asymmetry of the main peak and variability in the FWHM ratio of the bands located at 152 cm^−1^ and 1083 cm^−1^. The value of the ratio ranges from 2.3 to 2.83 with no apparent trend or rule. This is caused by the fact that the band at 152 cm^−1^ is a lattice mode, therefore its width is highly influenced by the orientation of the crystal. Also, discontinuities in crystal growth may affect the value of the ratio.

**Figure 3:**
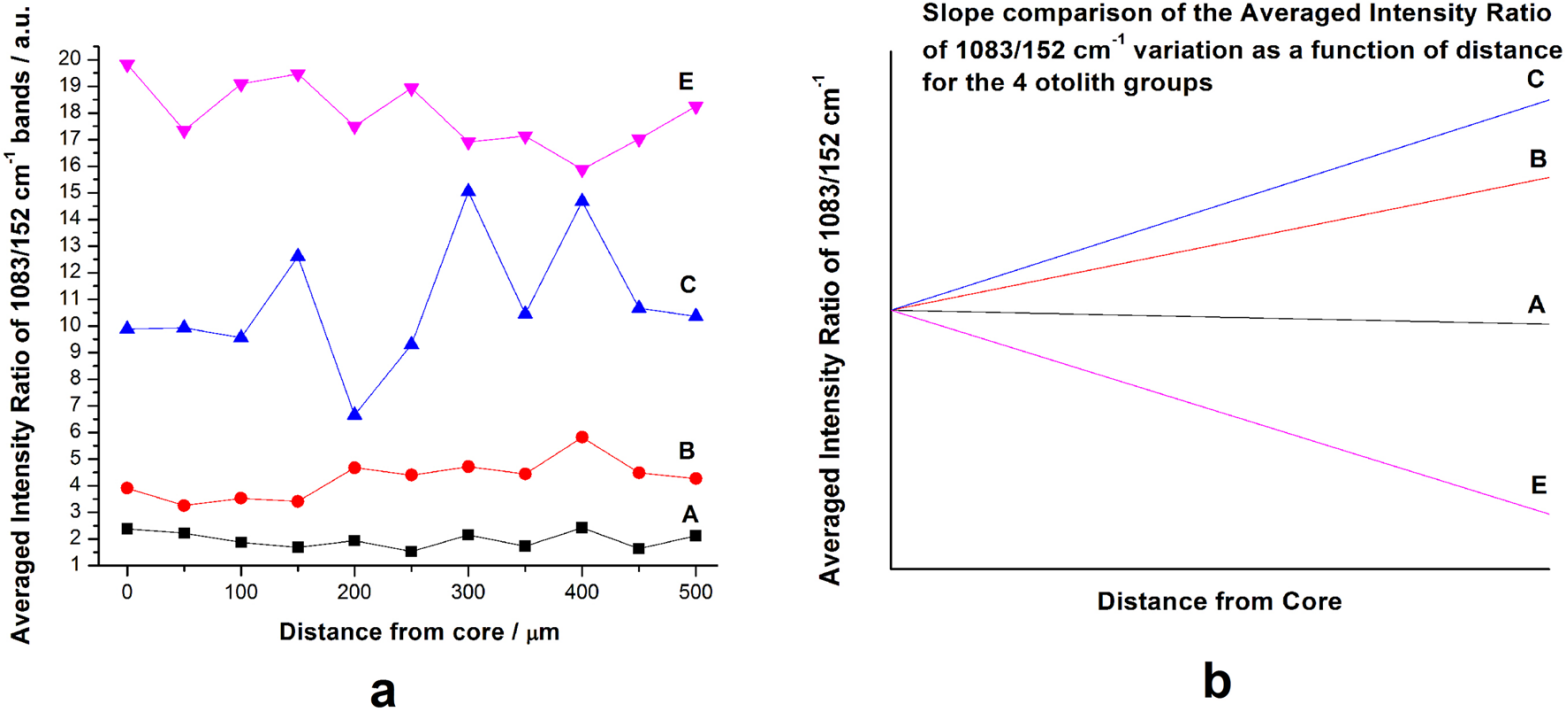
(**a**) Comparative evolution of the FWHM of the main Raman stretching mode of carbonate at 1083 cm^–1^ for the four groups of otoliths plotted against the distance from core. (**b**) Slope comparison of the FWHM variation of the 4 distinct groups after averaging and linear fitting.

The FWHM variation of the 1083 cm^−1^ band, collected from the core to the edge on a 500 µm line with a 50 µm step was averaged for every group and the results were plotted against the distance from the core (Fig. 3a). Although no clear pattern or variation model can be distinguished between the groups, the FWHM gradually decreases from the core to the margins in each case suggesting a tendency of increasing the crystallinity during fish lifetime. Each graph was subsequently linearly fitted, and the resulting straight lines are presented in Fig. 3b for comparison. In terms of slopes of the fitted lines, the four groups can be distinguished among themselves, with Groups A and B (aquaculture and coastal waters) having a significantly higher slope than Groups C and D (open sea and estuary). There was no correlation with FWHM in characteristic Raman band (cm^-1^) and mean monthly water temperature and salinity values implying that these parameters have little effect on Raman band shape from otolith spectrum.

**Figure 4:**
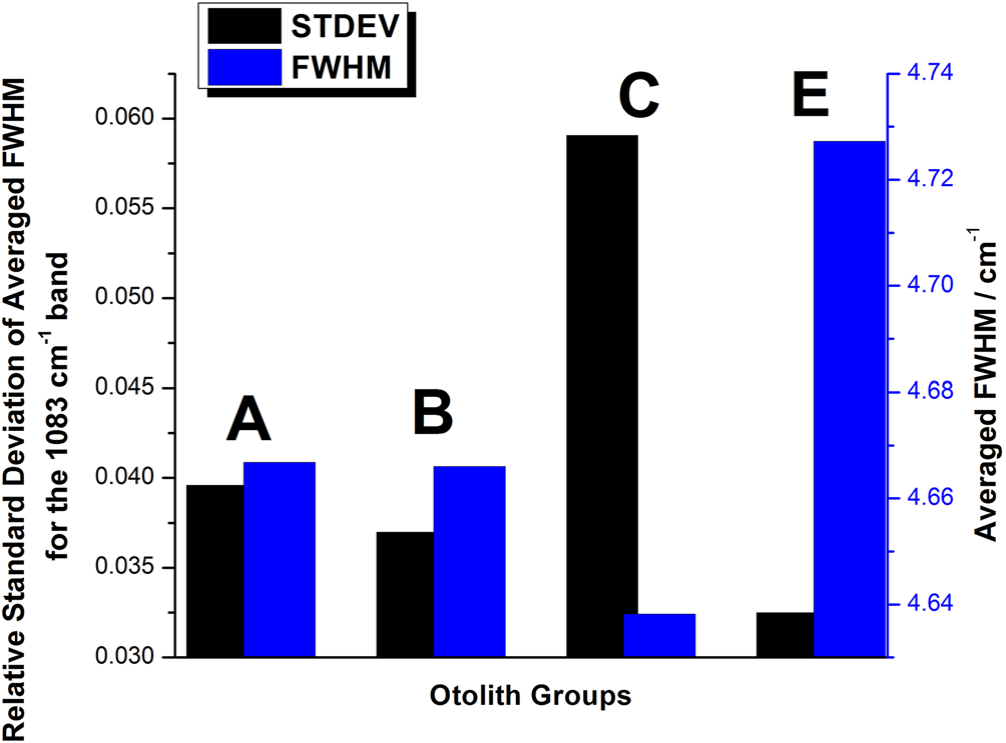
Relative Standard Deviation of the FWHM calculated for each otolith group. With blue, Group A, green Group B, red Group C and black Group E.

**Figure 5:**
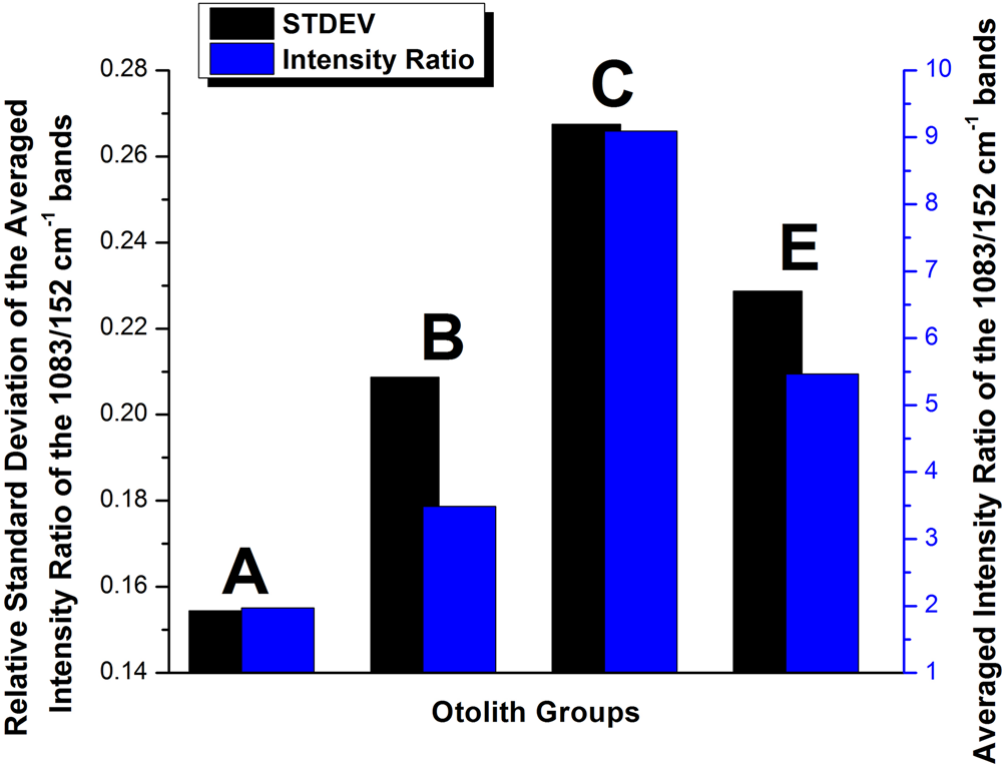
Relative Standard Deviation of the Intensity ratio of the 1083/152 cm^–1^ bands calculated for each otolith group. With blue, Group A, green Group B, red Group C and black Group E.

Furthermore, in order to determine the crystallinity variation across the lifetime of the otoliths, we calculated the Relative Standard Deviation (RSD) for each group separately, with the results being presented in Fig. 4. The RSD was calculated as the ratio of the Standard Deviation and the mean value for each group. In terms of standard deviation, the groups are relatively closely matched, except Group C (open sea), which is significantly higher than the other groups, 50% higher than Group A, the group with the next highest RSD (0.059 to 0.039). The RSD was again calculated for the previously presented Intensity ratios as well, with the results presented in Fig. 5. Unlike the RSD of the FWHM values, RSD for the intensity ratios presents a wider spread of values, with each group being differentiated. Moreover, just like in the case of the FWHM, the largest RSD was observed for the Group C set of values. This result further confirms that the crystal growth presents the largest variance for the open sea group. A strong correlation between crystallization and the intensity ratio of the 1083/152 cm^−1^ bands is to be expected as the 152 cm^−1^ lattice mode is strongly affected by the orientation of the aragonite crystals.

**Figure 6:**
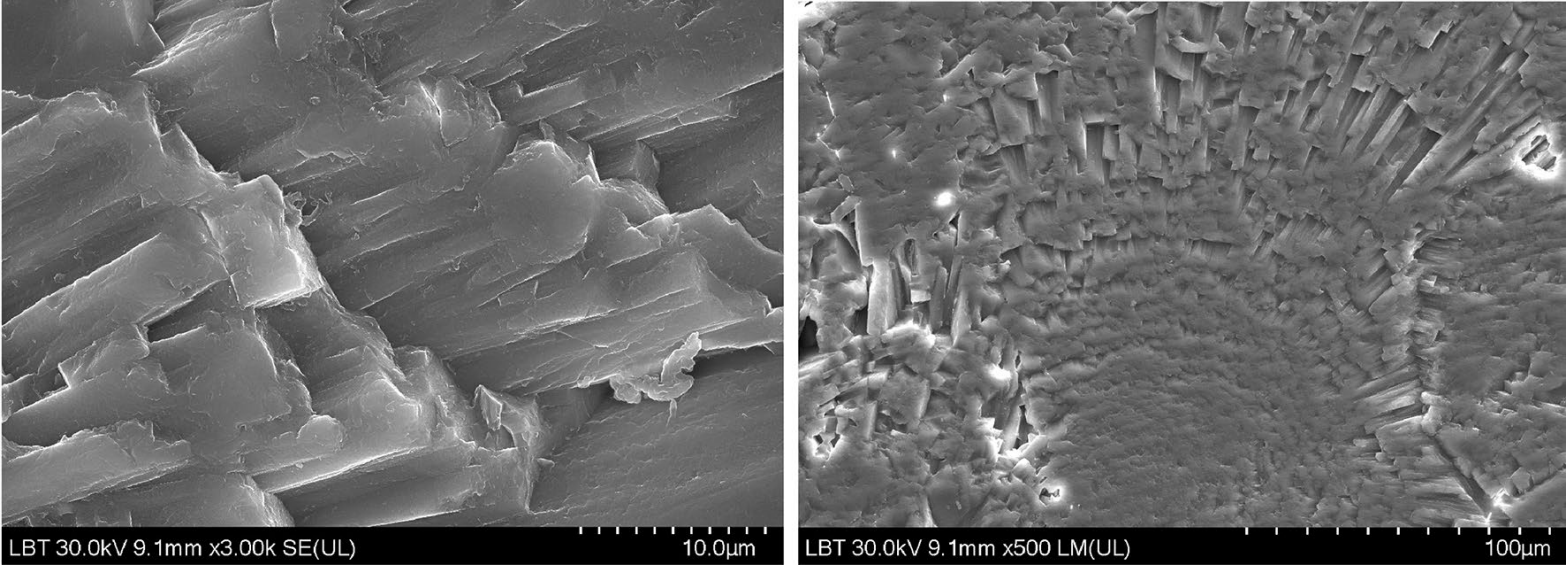
Scanning Electron Microscopy images of a randomly selected otolith surface with different magnifications, revealing the needle-like aragonite crystal structure (left) and the radial crystal growth (left).

**Figure 7:**
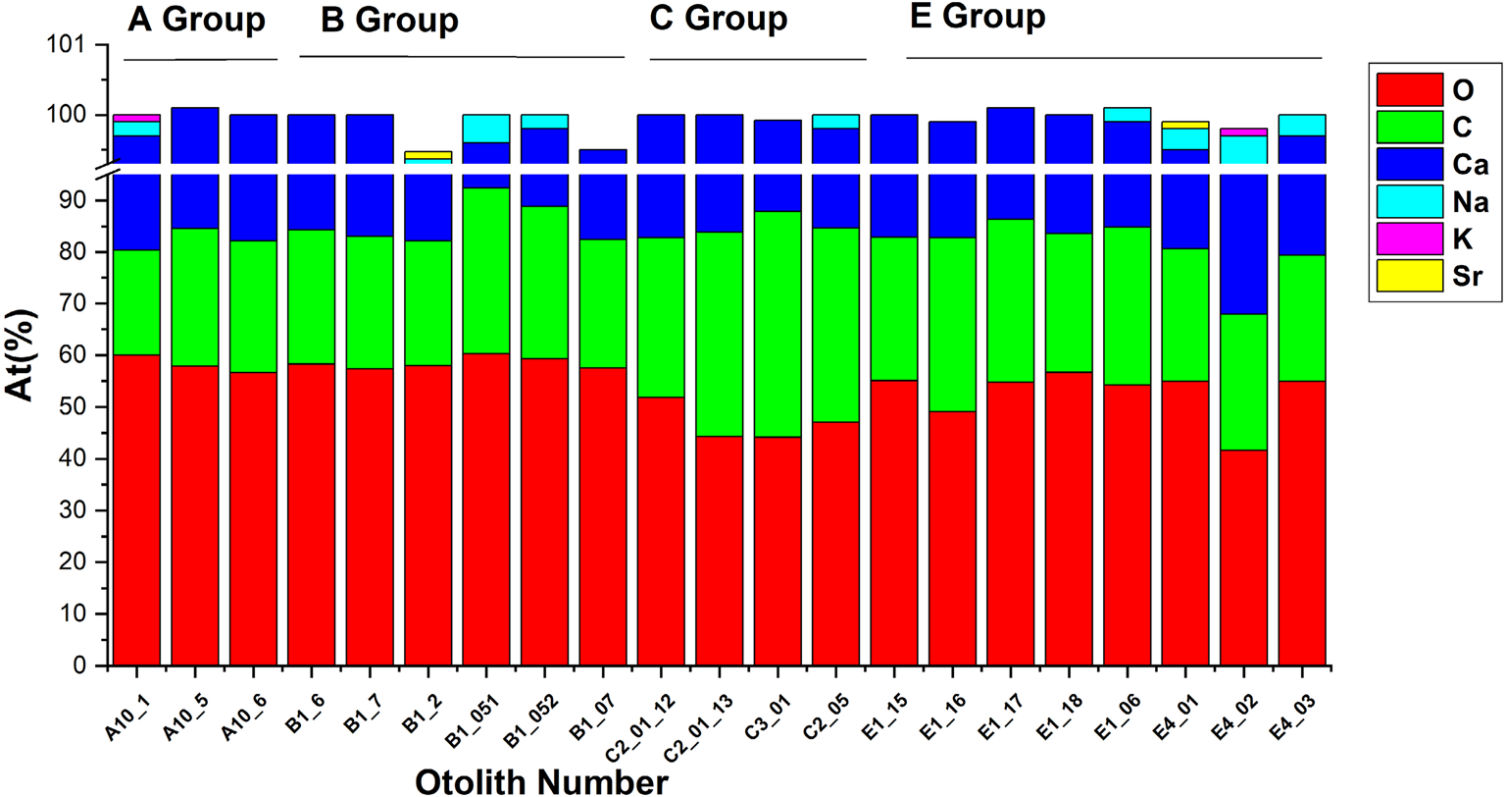
EDX comparative data on the four otolith groups showing the atomic weight At (%) plotted for O, C, Ca, Na, K and Sr.

Scanning Electron Microscopy (SEM) images were presented in order to further understand the microstructure of the otolith crystal (Figs. 6, 7). The SEM images reveal the crystalline structure of the aragonite as well as the growth patterns and discontinuities in growth from its surface. These discontinuities in growth, as well as the needle-like shape of the crystal observed in the images, are correlated with the Raman spectral data, the FWHM variation indicates variable crystallinity and discontinuities, along the growth line as well, while the position of the carbonate bands confirms the CaCO_3_ matrix polymorph as aragonite. Also, the energy-dispersive X-ray spectroscopy data further point out that there are no other major elements present in the otolith, except for the calcium carbonate matrix.

**Figure 8:**
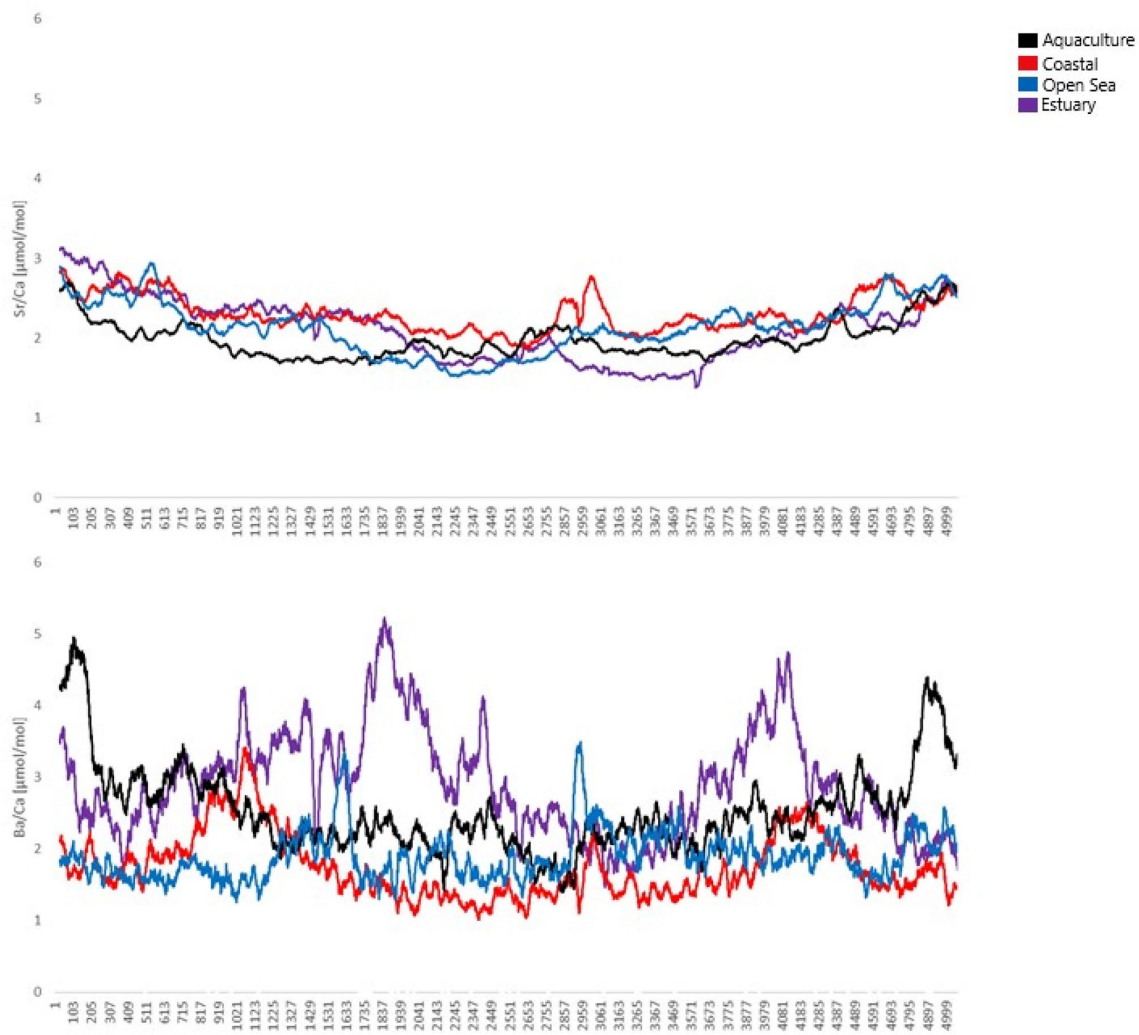

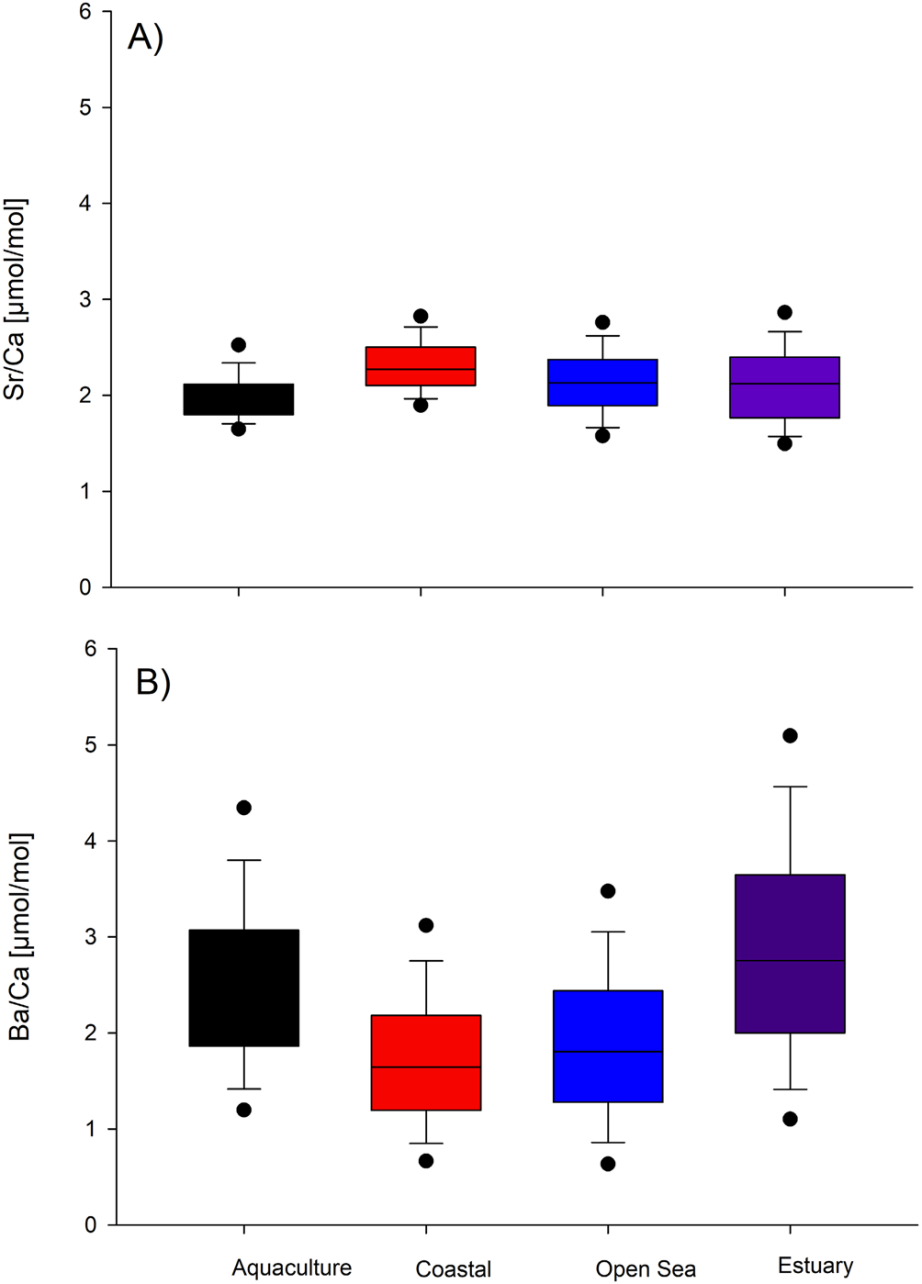
Box plots of median (± standard deviation) for (A) Sr/Ca; (B) Ba/Ca and Sr/Ca (C) and Ba/Ca (E) data series for all analyzed *Sparus aurata* specimens for 4 groups of interest: A—aquaculture, B—coastal area, C—open sea and E—estuary. Sampling conducted through the otolith core, along the axis of maximal growth. Results are displayed as 31-pt arithmetic running averages (x-axis).

**Figure 9:**
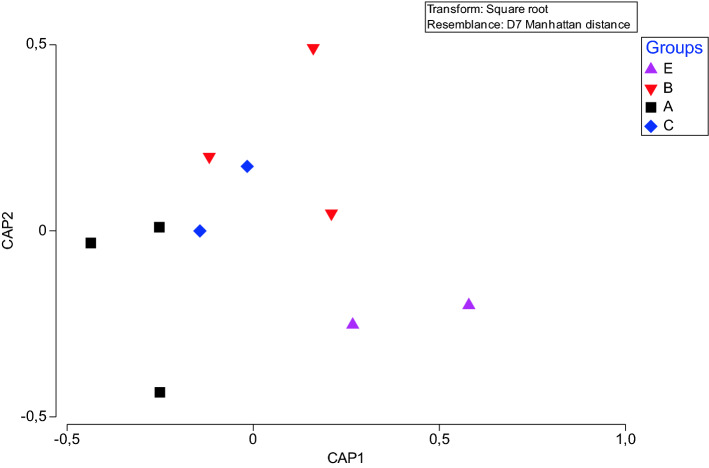
Canonical variate plot (CAP) for Sr/Ca element chemistry of the otoliths of *Sparus aurata* grouped specimens for A—aquaculture, B—coastal area, C—open sea and E—estuary.

Finally, according to LA-ICP-MS analysis, Ba/Ca and Sr/Ca varied within otoliths as well as between otoliths of fish collected from different areas (Fig. 8). Sr/Ca values were similar through the whole length of otolith growth with slightly increased ratios near the otolith core in samples collected from coastal waters (B) and the open seas (C). Ba/Ca values were varied within otolith length especially in samples collected from estuarine (E) waters and rearing environment (A). There were significant differences among sites for Sr/Ca (Pseudo-F = 1.550, p = 0.011) but not for Ba/Ca (Pseudo-F = 1.230, p = 0.124). The latter is well correlated with SEM–EDX data. Moreover, Ba was not detectable under SEM–EDX while Sr was spuriously present as showed in Fig. 7. A separate CAP analysis among four groups of interest gave successful discrimination just for Sr. In particular, Sr/Ca ratios were correctly allocated based on the otolith chemistry information to aquaculture group (66.7%) while other groups differed greatly (Fig. 9).

## Discussions

There is still a gap in the fundamental understanding of otolith biomineralization regarding biogenesis and crystal growth mechanisms of fish otoliths compared to the number of published investigations related to otolith use for age and growth estimation and habitat use pathways^44^. The knowledge about biochemical processes driving otolith macrostructure formation often remains incomplete, although its clearly acknowledged that there is a great variability at the individual, population, and species levels^45^. Opacity changes have been shown to depend on growth rate^45,46^, age and metabolism^47,48^ and sexual maturity^45,48^. Regarding environmental factors, low feeding conditions^49^ as well as temperature and photoperiod^45^ were shown to affect otolith opacity. There are a number of chemical analytical methods used to determine the chemical composition of otoliths and spectrometric methods to determine the shape, structure and crystallinity as well as those methods used to validate the periodicity of growth ring formation on hard structures^1,3–5,8^.

In the Adriatic Sea, open waters are oligotrophic ^50^, and unlike aquaculture rearing environments and more productive transitional waters, there is less food and organisms, and so *S. aurata* must forage and feed on different types of prey^38,40^. *Sparus aurata* underwent ontogenetic shifts in diet from zooplankton, selected by larvae, to mysids, errant polychaeta, amphipods and isopods for juveniles, and finally to hard prey (decapods, gastropods and bivalves) in adults^51^. Adults in open sea prey more on decapods where bivalves are less available by moving away from the coast). Without doubt, diet selection varies at *S. aurata* with species and environments as an ecosystem process^51^. However, as mentioned above, open sea waters are oligotrophic ^50^, but they are less variable in oceanographic factors than transitional, estuarine waters^52^. Understanding the effects of environmental factors and physiological processes related to ontogeny and metabolism on the chemical composition and structure of otoliths could be very useful in the field of fisheries science^53^. Otolith usage in reconstructing fish migratory pathways and connectivity between populations and stocks or in investigating population structure could prove to be very important in the future fishery related issues. Since some elemental ratios in the otoliths like those of Sr/Ca and Ba/Ca were linked to concentrations of Sr and Ba in ambient water and salinity, a higher Sr/Ca suggest marine and higher Ba/Ca freshwater environment^20,54^. Thus, Sr and Ba are usually used for successful reconstruction of environmental and coastal-estuary migration pathways for individual fish^19,55^.

Results of present study on the crystal structure of otoliths in *S. aurata* in four different environment via CRM have been confirmed by previous studies^27^.The initial increase corresponding to first spring–summer period and the period of most intensive growth due to higher temperature and longer photoperiod, then decrease of the FWHM from the early stages of life can be related to the specific biomineralization processes^12^ as well as environmental factors^13^ (growth become slower in first winter due low temperature). However, no significant correlation with FWHM and environmental factors were obtained suggesting that slope of the FWHM variation that distinguished aquaculture and costal groups from open sea and transitional, estuarine waters can be related to prey availability^47–49^. Moreover, open marine waters as potentially different environment in term of conducted analysis are proved by RSD of the FWHM. In terms of the factors affecting the growth and biomineralization of the otoliths, this result is somewhat expected as the individuals from Groups C have spent most of their lifetime in the open Sea. Compared to the other groups, the open sea provides an environment with no constrictions^50^, and free of human control, with a large number of external (mostly prey and predator relationships, since oceanographic feature are there less variable than in the estuarine areas) factors affecting the growth and crystallization of the otoliths^3,12,13^. SEM clearly confirmed CRM findings without bringing in new information of *S. aurata* otolith composition. Finally, Ba/Ca ratios obtained by LA-ICP-MS were also indicative of the separation of groups associated with farming and transitional waters. Since no statistically significant difference related to environmental variables and Sr/Ca and Ba/Ca ratios were found, and both groups can be recognized as eutrophicated environments^50^, it can be hypothesized that foraging and feeding, i.e. the amount and availability of prey in specific environment are possible causes of observed changes in the chemical composition of *S. aurata* otoliths^29–32,34^.

There are a number of potentially influencing factors that may have affected the results in the present study, from the sampling itself, the selection of individuals in the respective length class, the complex preparation of otoliths for each of three method conducted and finally the known shortcomings of each method^3^. According to the theory the Raman band shape is influenced by environmental factors such as temperature and pressure^56^, by the internal properties of the molecules such as bond shape and length, spatial symmetry as well as the presence or absence of spatial order^26,27^ and by the optics of the spectrometer^12^. In the case of solids, the band broadening, going from crystals to amorphous solids is due to the ideal spatial organization and symmetry of the former and lack of any spatial organization of the latter^57,58^. Moreover, a band can be narrowed by instruments with better optics which eliminate the spectra of underlying layers as well as the errors caused by the irregularity of the probe’s surface^59^. Regarding the experimental parameters, the spectra were acquired in the same conditions, thus they should not cause any differences in the width of the bands. Also, additional samples and stable isotopes analyzes would allow better identification of causal relationships. However, the use of Sr stable isotopes for broader ecological questions required larger sample sizes to describe nursery habitats use, metapopulation dynamics, and stock discrimination similar to studies oriented just on otolith geochemical composition^57,58^. Unfortunately, studies are usually flawed due to lack of water and sediment samples which makes it impossible for reliable comparison and calculation of relationship between water and otolith elemental chemistry. For sure, such limitations have to be consider in future sampling designs. Although, sampling on larger length class will give valuable opportunity to test the individual impact of growth on the structure of otoliths particularly if feeding intensity is potentially one of key factor in structuring otoliths and thus enabling their allocation into different environments.

Confocal Raman micro-spectrometry offers a non-destructive alternative which can provide valuable information on the otolith composition and crystallinity. The Full Width at Half Maximum parameter’s unique feature of reflecting the crystallinity and polymorphism combined with other environmental parameters including feeding and foraging has the potential of accurately linking the individuals with specific areas, during specific periods of fish path during lifetime, and not just validating the periodicity of rings formation as usually considered^3^. The FWHM variation during lifetime does not follow any mathematical formula, however, this is expected due to the large number of factors which have an influence on the complex otolith growing process. Due to the influence of the environmental factors as well as that of the internal biomineralization processes on growth, it is likely that the apparent trend of the FWHM observed in the present study may not appear in otoliths of other species from other geographic areas. Further studies are certainly necessary to confirm any correlation found between environmental change and otolith characteristics during the fish lifetime and migration path. The absence or presence of vaterite in the calcium carbonate matrix is another factor that affects the growing process, thus the specific cycle in FWHM variation observed on this individual may not appear in otoliths containing significant vaterite fractions. The intensity of the Raman bands, may also reflects the daily activity of individual fish in foraging for prey or searching for shelter, as shown by the Raman mapping on the daily growth rings in previous work^27^. The alternation of high and low intensities in coincidence with the rings clearly depict the activity on a micro-scale with high intensities corresponding to larger mineral deposits. Daily rings show discontinuities in growth the same as annual rings do, as proven by the scanning electron microscopy images, thus indicating that the otolith growing processes on a micro-scale resemble those on a macro-scale. Moreover, the age of the fish also affects the results of the measurements, since an older individual has a larger number of growth cycles, and thus can provide with a more detailed picture of the variation of crystallinity during its lifetime.

Finally, results of present study show the additional potential for Raman spectroscopy, not just for validation of annual ring formation but also as a complementary tool for inference of migration or origin of fish based on otolith composition and structure like other well-established technique, like LA-ICP-MS. The results of all complementary methods show that the least variable results were obtained from aquaculture otoliths group which refers to the rearing environment, probably due to the fact that the conditions of the that environment and food availability are controlled^60^. On the other hand, the highest variability is related to open marine water and coastal groups, probably due to the oligotrophic nature of these waters within Adriatic Sea. At the end, high-productivity estuarine waters showed a specific footprint recognized by all three methods. In future works, to accurately determine the cause-and-effect relationships, water samples from all groups should be included for screening optimization to see how much the water signals have been modified by physiological mediation for Raman band width, bioaccumulation, crystallinity, or elemental composition of *S. aurata* otoliths in different environments since in more complex organisms, physiological processes can significantly influence ion transport, binding and availability for incorporation into calcifying structures^61^.

## Materials and methods

### Otolith sampling and preparation

As described in our preliminary paper^27^ the sampling details for one otolith of similar origin, in this study we comprised 16 adults of gilthead seabream *Sparus aurata*, aged 2–3 years (total length 22.8–28.5 cm, total weight 140.9–325.0 g), collected on four locations in the eastern Adriatic during 2017: A (aquaculture rearing cages), B (coastal waters), C (open sea) and E (estuary). Environmental data in term of mean water temperature and salinity for each sampling group were provided^62^. In addition to biometric measures, otoliths have been extracted, rinsed with distilled water, dried, and weighed. For standardization purposes, the left sagittae were systematically studied. The otoliths were washed in 30% hydrogen peroxide solution for 2–4 min and rinsed in distilled water. Samples were then cleaned in an ultrasonic bath (SONOREX SUPER RK 103 H) for 2 min and left to air-dry.

Otolith preparation was done following protocol that have been already described in the already mentioned paper^27^. Epoxy resin (MEGAPOXY H) was prepared by mixing three parts of resin and one part of hardener. These two components were stirred together for 2–3 min until the mixture changed color to translucent. The otoliths were embedded in the molds which were lightly coated with Struers Silicone and they were left to dry in the fume cupboard for one day. Isomet low-speed diamond bladed saw was used for preparing otolith sections. Saw was fitted with two blades separated by a spacer (500 µm), producing a 400–500 µm otolith thin section. Each thin section was carefully grounded with Struers Labopol-5 using Struers wettened silicon carbide paper (4000 grit) at the speed of 50 rpm. Thin sections were then polished using a soft cloth sprayed with diamond paste (3 µm) and washed again in ultrasonic bath for 2 min.

Series of images for every otolith were taken using ZEISS microscope equipped with AXIO camera and ZEN 2 (blue edition) program. Photos were then stitched together with Image-Pro Premier 9.1 software to obtain one composite image for each thin section. Core and growth marks sections were observed under an optical microscope. Opacity data were acquired on transects from the core to the ventral edge with black areas corresponded to opaque zones.

### Confocal Raman spectra otolith analysis and data processing

Confocal Raman spectra were acquired using a Renishaw InVia Confocal Raman System with a Cobolt DPPS laser emitting at 532 nm. During Raman microscopy, as already described^27^, three different objectives were used: 5 × (NA 0.12), 20 × (NA 0.35) and 100 × (NA 0.9) respectively. For single spectra acquisition at controlled distances from otolith core, the acquisition parameters were 1 s, 1 acquisition, 200 mw laser power. An edge filter has been employed to record spectra in the 90–1840 cm^−1^ spectral range with 0.5 cm^−1^ resolution^27^. A Rencam CCD was used for signal detection while data acquisition has been achieved with WIRE 3.4. Micrographs of the morphological details have been acquired along with spectral data acquisition using the video image facility of the WIRE software^27^. Before Raman measurement, annual growing rings have been detected via optical microscopy. Also, daily rings have been established and mapped using the univariate signal-to-baseline criteria of the acquisition software.

All the spectra were processed using OriginPro 9.1 software. In order to investigate the differences between the otoliths with different origins: Aquaculture (Group A), Coastal Waters (Group B), Open Sea (C) and Estuary (E); we studied the FWHM variation of the bands in Raman spectra collected from the core to the edge. The spectra were baseline corrected and then were fitted with a Gauss function, using the Quickfit option, with adjusted r-square (coefficient of determination) ranging from 1 to 0.992 for group A, from 0.999 to 0.958 for Group B, from 1 to 0.934 for Group C and from 1 to 0.93 for Group E. The position of the 1083 cm^−1^ band measured in ten points from the center to the edge of the otolith (with a 50 μm step), presented a standard deviation of 0.1 cm^−1^ for group A, 0.12 cm^−1^ for Group B, 0.18 cm^−1^ for Group C and 0.14 cm^−1^ to for Group E. The positions of the Raman band at 1083 cm^−1^ for the centers of the otoliths recorded on four samples from each group, presented standard deviation of 0.194 cm^−1^ for group A, 0.061 cm^−1^ for group B, 0.137 cm^−1^ for group C and 0.057 cm^−1^ for group E. All standard deviations were performed using the standard deviation function in Microsoft Excel 2010.

### SEM–EDX otolith analysis

A SU8230 Hitachi ultra-high resolution cold-field emission scanning electron microscope have been used for data acquiring. The combination of topographical and compositional information at a feature resolution of up to 1 nm in optimal conditions can be obtained by using this instrument. Firstly, otolith sections were dried in oven at 40 °C. After drying, samples were adherently placed on Hitachi stub SEM holder (aluminum holder with M4 threads covered with carbon discs of 3 mm thickness). A Quorum Q150T sputtering sample coater providing gold sputtering of 10 nm thickness for high resolution imaging. Also, evaporating carbon for EDX analysis was employed. An Oxford energy-dispersive x-ray module (Oxford, UK, AZtec Software) was used for elemental detection.

### LA-ICP-MS analysis of otoliths and data processing

Element concentrations of the otoliths were determined by LA-ICP-MS in line scan mode, along the axis of maximal growth, through the otolith core. Analyses were performed at the Institute of Geosciences, JGU, Mainz, Germany, using an ESI NWR193 ArF excimer laser ablation system equipped with the TwoVol2 ablation cell, operating at 193 nm wavelength, coupled to an Agilent 7500ce quadrupole ICP-MS. Sample surfaces were preablated prior to each line scan to prevent potential surface contamination. The laser repetition rate was 7 Hz and laser energy on samples was about 3 J/cm^2^. Background intensities were measured for 15 s. Line scans were carried out at a scan speed of 5 μm/s, using a rectangular beam of 50 × 40 µm (preablation beam 80 × 40 μm). Monitored isotopes included ^7^Li, ^23^Na, ^26^Mg, ^43^Ca, ^55^Mn, ^66^Zn, ^88^Sr, ^97^Mo, ^137^Ba, ^138^Ba, ^208^Pb and ^238^U. Signals were monitored in time-resolved mode and processed using an in-house Excel spreadsheet^66^. Details of the calculations are given in^67^. The concentration of Ca as an internal standard in otoliths was taken as 38.8% by weight or 388,000 µg/g following the determination of otolith Ca concentration^68^. Synthetic glass NIST SRM 612 (National Institute of Standards and Technology) was used to calibrate element concentrations of otolith samples and quality control materials (QCMs; USGS MACS-3, USGS BCR-2G, NIST SRM 610) were used to monitor accuracy and precision of the LA-ICP-MS analysis and calibration strategy applying the preferred values available from the GeoReM database (http://georem.mpch-mainz.gwdg.de/, application version 26; compare also^63–65^. Element concentrations determined for the QCMs agreed within uncertainties with the preferred values of GeoReM. Concentrations determined on otoliths were converted to molar concentrations and standardized to calcium.

Element-to-Ca data for 10 elements were determined for all specimens. Ba/Ca and Sr/Ca ratios were above the detection and quantification limits and thus those elements were included in further analysis. For data visualization, element linear raster was smoothed using a 31-pt arithmetic running average. To enable comparison between samples and sites and to eliminate potential confounding effects due to temporal variation in the factors influencing otolith chemical composition, all otoliths were reduced on a raster line length of the smallest sample^69^. Statistical analysis was done using PRIMER (V. 7.0.13; Auckland, NZ^70^. Differences in otolith elemental composition were evaluated via the permutational analysis of variance (PERMANOVA) using Manhattan distance dissimilarity matrices. Canonical analyses (CAP) was used to estimate the accuracy of otolith element signatures in classifying fish to their collection site.

## Data Availability

The datasets used and/or analyzed during the current study available from the corresponding author on reasonable request.
